# Boosting photosynthesis opens new opportunities for agriculture sustainability and circular economy: The BEST‐CROP research and innovation action

**DOI:** 10.1111/tpj.17264

**Published:** 2025-02-05

**Authors:** Paolo Pesaresi, Pierre Bono, Stephane Corn, Cristina Crosatti, Sara Daniotti, Jens Due Jensen, Ivo Frébort, Eder Groli, Claire Halpin, Mats Hansson, Goetz Hensel, David S. Horner, Kelly Houston, Ahmed Jahoor, Miloš Klíma, Hannes Kollist, Clément Lacoste, Boubker Laidoudi, Susanna Larocca, Caterina Marè, Nicolas Le Moigne, Chiara Mizzotti, Tomas Morosinotto, Klaus Oldach, Laura Rossini, Sebastian Raubach, Miguel Sanchez‐Garcia, Paul D. Shaw, Rodolphe Sonnier, Alessandro Tondelli, Robbie Waugh, Andreas P.M. Weber, Dmitry Yarmolinsky, Alessandro Zeni, Luigi Cattivelli

**Affiliations:** ^1^ Department of Biosciences University of Milan Milan 20133 Italy; ^2^ FRD‐CODEM (Fibres Recherche Développement‐Construction Durable et EcoMatériaux), Hôtel de Bureaux Technopole de l'Aube en Champagne 2 rue Gustave Eiffel, CS 90601 Troyes Cedex 9 10 901 France; ^3^ LMGC, IMT Mines Ales Univ Montpellier, CNRS Alès France; ^4^ Council for Agricultural Research and Economics (CREA) – Research Centre for Genomics and Bioinformatics Fiorenzuola d'Arda 29017 Italy; ^5^ Consorzio Italbiotec Piazza della Trivulziana 4 Milan 20126 Italy; ^6^ Nordic Seed A/S Kornmarken 1 Galten 8464 Denmark; ^7^ Czech Advanced Technology and Research Institute (CATRIN) Palacký University Olomouc Šlechtitelů 27 Olomouc 783 71 Czech Republic; ^8^ S.I.S. Società Italiana Sementi via Mirandola di Sopra 5, 40068 S. Lazzaro di S Bologna Italy; ^9^ Division of Plant Sciences, School of Life Sciences University of Dundee at the James Hutton Institute Dundee DD2 5DA UK; ^10^ Department of Biology Lund University Lund 22362 Sweden; ^11^ Cluster of Excellence in Plant Sciences “SMART Plants for Tomorrow's Needs” Heinrich Heine University Düsseldorf Düsseldorf Germany; ^12^ Centre for Plant Genome Engineering Heinrich Heine University Düsseldorf Düsseldorf Germany; ^13^ Cell and Molecular Sciences James Hutton Institute Errol Road, Invergowrie Dundee DD25DA UK; ^14^ Úsovsko a.s. Klopina 33 Klopina 789 73 Czech Republic; ^15^ Institute of Bioengineering University of Tartu Tartu 50411 Estonia; ^16^ Institute of Plant Sciences Paris‐Saclay (IPS2) Université Paris‐Saclay, CNRS, INRAE Université Evry, Université Paris Cité Gif sur Yvette 91190 France; ^17^ Polymers, Composites and Hybrids (PCH) IMT Mines Ales Ales France; ^18^ SO.G.I.S. Industria Chimica SpA Cremona Italy; ^19^ Department of Biology University of Padova Padova Italy; ^20^ KWS LOCHOW GmbH Bergen Lower Saxony Germany; ^21^ Department of Agricultural and Environmental Sciences–Production, Landscape, Agroenergy (DiSAA) University of Milan Milan 20133 Italy; ^22^ Information and Computational Sciences James Hutton Institute Errol Road, Invergowrie Dundee DD2 5DA UK; ^23^ International Center for Agricultural Research in the Dry Areas (ICARDA) Rabat 10100 Morocco; ^24^ Institute for Plant Biochemistry Heinrich Heine University Düsseldorf Düsseldorf Germany

**Keywords:** barley, canopy photosynthesis, straw quality, circular bioeconomy, straw‐based panels, composites, biolubricants, feed

## Abstract

There is a need for ground‐breaking technologies to boost crop yield, both grains and biomass, and their processing into economically competitive materials. Novel cereals with enhanced photosynthesis and assimilation of greenhouse gasses, such as carbon dioxide and ozone, and tailored straw suitable for industrial manufacturing, open a new perspective for the circular economy. Here we describe the vision, strategies, and objectives of BEST‐CROP, a Horizon‐Europe and United Kingdom Research and Innovation (UKRI) funded project that relies on an alliance of academic plant scientists teaming up with plant breeding companies and straw processing companies to use the major advances in photosynthetic knowledge to improve barley biomass and to exploit the variability of barley straw quality and composition. We adopt the most promising strategies to improve the photosynthetic properties and ozone assimilation capacity of barley: (i) tuning leaf chlorophyll content and modifying canopy architecture; (ii) increasing the kinetics of photosynthetic responses to changes in irradiance; (iii) introducing photorespiration bypasses; (iv) modulating stomatal opening, thus increasing the rate of carbon dioxide fixation and ozone assimilation. We expect that by improving our targeted traits we will achieve increases in aboveground total biomass production without modification of the harvest index, with added benefits in sustainability via better resource‐use efficiency of water and nitrogen. In parallel, the resulting barley straw is tailored to: (i) increase straw protein content to make it suitable for the development of alternative biolubricants and feed sources; (ii) control cellulose/lignin contents and lignin properties to develop straw‐based construction panels and polymer composites. Overall, by exploiting natural‐ and induced‐genetic variability as well as gene editing and transgenic engineering, BEST‐CROP will lead to multi‐purpose next generation barley cultivars supporting sustainable agriculture and capable of straw‐based applications.

## Significance Statement

BEST‐CROP exemplifies the integration of agricultural innovation and industrial sustainability, setting a transformative path for future crop production and biomass utilization, while promoting circular economy principles. By boosting total aboveground biomass production without altering the harvest index, the project aims to develop next‐generation, multi‐purpose barley cultivars with improved photosynthesis and greenhouse gas assimilation, making straw viable for biolubricants and alternative feed sources, and enabling straw‐based construction materials and polymer composites.

## INTRODUCTION

The continuous growth of the world population is driving an increase in the demand for food and feed up to 50% by 2050 (van Dijk et al., 2021). This, in turn, will require a boost of agricultural productivity, while simultaneously reducing the negative environmental impacts (Haughey et al., 2023), like freshwater consumption and greenhouse gas (GHG) emissions, and without increasing cultivated land area.

BEST‐CROP (https://www.bestcrop.eu)—Boosting photosynthESis To deliver novel CROPs for the circular bioeconomy—is a research and innovation action aiming to contribute to these challenges in the frame of the circular and bioeconomy transition, supporting the European Green Deal. Specifically, BEST‐CROP targets barley (*Hordeum vulgare* L.), a major crop worldwide, with 153.6 million tons of grain produced in the 2022/23 season and an almost equivalent amount of straw covering around 60 million ha of world arable land (Taner et al., 2004).

**Figure 1:**
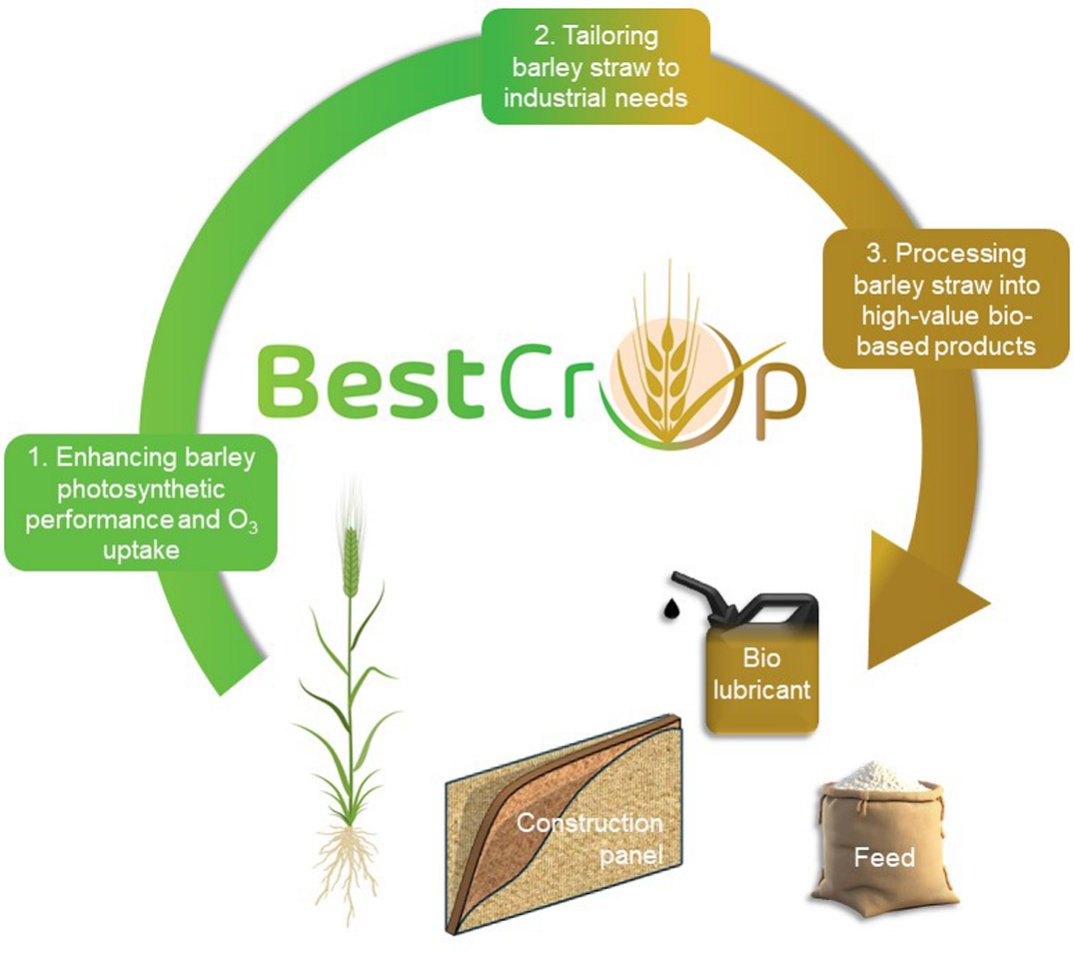
Overview of the main objectives and products of BEST‐CROP. Next generation barley plants with optimized canopy photosynthesis will provide high‐quality straw to produce biolubricants, construction panels, and feed.

The ambition is to develop next generation multi‐purpose barley plants with: (i) increased uptake of carbon dioxide (CO_2_) and ozone (O_3_); (ii) enhanced total biomass production without modification of the harvest index; (iii) straw composition tailored for transformation into high‐value bio‐based industrial products for the feed, green chemistry (biolubricants), construction, and composites sectors (see Figure 1).

The experimental plan builds on evidence that genetic variability impacting photosynthesis (Croce et al., 2024; Flood et al., 2011), canopy architecture (Mantilla‐Perez & Salas Fernandez, 2017; Sakamoto et al., 2006; Shaaf et al., 2019), and barley straw composition, in terms of lignin (Daly et al., 2019; Grove et al., 2003; Halpin, 2019; Li et al., 2003; Zhang et al., 2010) and protein content (Bellucci et al., 2017; Przulj & Momcilovic, 2001; White et al., 1981), exists within barley germplasm and mutant collections and can be exploited to design a next generation barley plant. Research conducted in different laboratories worldwide has demonstrated that genetic manipulation of model organisms has the potential to improve photosynthesis efficiency (for a review see Croce et al., 2024), O_3_ uptake (Brosché et al., 2010; Morales et al., 2021; Sierla et al., 2018; Yamauchi et al., 2016), and straw quality (Araus et al., 2016; Guo et al., 2020; Halpin, 2019; Karunarathne et al., 2022; Wang, Nian, et al., 2018). The performance of advanced breeding lines, carrying the selected traits, will be evaluated under field conditions in different European agroclimatic scenarios by monitoring, among other things, total biomass production, grain yield, and O_3_ uptake. Finally, BEST‐CROP tailors the straw composition to different industrial transformation processes by increasing protein content for feeding insects capable of transforming straw into animal feed and biolubricants, as well as by modulating (up and down) the lignin content to make barley straw suitable to produce mycelium‐based construction panels (Alaneme et al., 2023), structural straw board (Amziane & Collet, 2017; Li et al., 2023; Tlaiji et al., 2022), and sandwich panels, as well as polymer composites (Bourmaud et al., 2018; Mohanty et al., 2018).

During more than one century of barley/wheat (*Triticum* ssp.) breeding, straw traits have been selected mainly to maximize yield and minimize lodging (semidwarf plants), while no attention has been given to straw composition. Although some straw transformation processes have been proposed, straw composition has never got attention from breeders and the current straw composition might not be the most suitable for every possible transformation. By tailoring straw composition to specific industrial needs, the project innovations will contribute to replacing non‐renewable with renewable resources. The production of chemical lubricants has a high environmental impact that could be reduced by substituting oil‐derived lubricants with biodegradable alternatives. Moreover, transforming straw into high‐nutrition feed using insects could reduce the need for dedicated protein crops. The increase in root biomass and the use of straw as raw material in the construction and composite sector promote carbon sequestration contributing to mitigate the effects of climate change. By shifting to utilizing straw‐based materials which are locally and widely available, BEST‐CROP will aid in decarbonizing relevant industries and will contribute to reducing their environmental impact.

These objectives will be driven forward based on highly innovative biotechnology approaches that exploit natural‐ and induced‐genetic variation, gene editing and genetic engineering techniques for improving the photosynthetic efficiency, and building a next generation barley plant that could be exploited directly in breeding programs, while also serving as proof‐of‐concept of gene function.

## OPTIMIZATION OF CANOPY PHOTOSYNTHESIS PERFORMANCE

The efficiency of solar energy conversion into biomass, during photosynthesis, is surprisingly low in crops, typically below 1% in temperate climates. For this reason, numerous strategies have recently been proposed to improve overall photosynthetic efficiency under different growth conditions (Kromdijk et al., 2016; Simkin et al., 2015, 2019; Slattery & Ort, 2019, 2021; Smith et al., 2023; South et al., 2019). These studies have explored various innovative approaches, from enhancing light harvesting to introducing new biochemical pathways aimed at increasing carbon fixation efficiency. In synergy with other EU‐funded projects such as CAPITALISE (https://www.capitalise.eu/), GAIN4CROPS (http://gain4crops.eu/) and PhotoBoost (http://www.photoboost.org/), BEST‐CROP aims to exploit promising strategies, selected based on proof‐of‐concept in *Arabidopsis thaliana* (L.) Heynh., tobacco (*Nicotiana tabacum* L.) and a few crops, demonstrating that photosynthesis can be readily improved and translated into breeding programs (Table 1).

**Table 1:** Main proof‐of‐concepts, demonstrating that photosynthesis can be readily improved, inspiring BEST‐CROP experimental plan

Optimization of canopy photosynthesis				
Trait	Target gene(s)	Tested species	Gain	References
*Altering the composition of the thylakoid electron transport chain*				
Reduced antenna size	Downregulation of the *43‐kDa chloroplast Signal Recognition Particle* (*cpSRP43*)	*Nicotiana tabacum* L.	Plants with 10% greater leaf‐to‐stem ratio, and 8.2% more canopy biomass accumulation under high‐density cultivation conditions	Kirst et al. (2018)
	A premature stop codon in the *43‐kDa chloroplast Signal Recognition Particle* (*cpSRP43*)	*Hordeum vulgare* L.	A 50% reduction in the chlorophyll content of leaves does not cause any penalty on total biomass production and grain yield under standard field conditions	Rotasperti et al. (2022)
Faster NPQ relaxation	Overexpression of *Violaxanthin DE‐epoxidase* (*VDE*), *Zeaxanthin Epoxidase* (*ZEP*), and the *PsbS* subunit of Photosystem II (PSII)	*N. tabacum* L.	15% greater plant biomass production in natural field conditions	Kromdijk et al. (2016)
	Overexpression of *Violaxanthin DE‐epoxidase* (*VDE*), *Zeaxanthin Epoxidase* (*ZEP*), and the *PsbS* subunit of Photosystem II (PSII)	*Glycine max* (L.) Merr.	In replicated field trials, seed yield increased by up to 33%	de Souza et al. (2022)
Additional electron sinks	Overexpression of *Flavo‐di‐iron proteins FlvA‐FlvB* from *Physcomitrium patens*	*Arabidopsis thaliana* L.	Protect photosystems under fluctuating light	Yamamoto et al. (2016)
	Overexpression of *Flavo‐di‐iron proteins FlvA‐FlvB* from *Physcomitrium patens*	*Oryza sativa* L.	Increased resistance to PSI photodamage under fluctuating light	Wada et al. (2018)
	Overexpression of cyanobacterial *Flavo‐di‐iron proteins Flv1/Flv3*	*A. thaliana* L.	Plants with 10–30% higher shoot dry weight	Tula et al. (2020)
	Overexpression of *Synechocystis Flavo‐di‐iron proteins Flv2‐Flv4*	*A. thaliana* L.; *N. tabacum* L.	Plants with increased tolerance toward high irradiation, salinity, oxidants, and drought	Vicino et al. (2021)
Photorespiration bypasses	Introduction of glycolate catabolic pathway from *Escherichia coli*	*A. thaliana* L.	Plants with at least 50% more root and shoot biomass, faster growth and more soluble sugars	Kebeish et al. (2007)
	Introduction of three alternative glycolate catabolic pathways in combination with the downregulation of the *Plastidal glycolate/glycerate translocator 1* (PLGG1)	*N. tabacum* L.	Plants with up to 24% increased biomass accumulation and improved light‐use efficiency of photosynthesis by 17%	South et al. (2019)
	Introduction of a catabolic pathway allowing complete oxidation of glycolate into CO_2_ catalyzed by three rice enzymes, that is, glycolate oxidase, oxalate oxidase, and catalase	*O. sativa* L.	Plants with significant increases in photosynthesis efficiency, nitrogen content, and up to 35% more biomass production	Shen et al. (2019)
Excluding stomatal limitation	Overexpression of the plasma membrane H^+^‐ATPase 1 (*OSA1*)	*O. sativa* L.	Plant with a 33% increase in grain yield and a 46% increase in N use efficiency	Zhang et al. (2021)
*Modifying canopy architecture*				
More upright leaves	Loss of function mutant in *DWARF4* gene encoding a cytochrome P450, CYP90B1, involved in brassinosteroid biosynthesis	*O. sativa* L.	Plants with more erect leaf angle, increased aboveground biomass, and grain yield at high planting densities.	Sakamoto et al. (2006)
	Loss of function mutant in *Leaf Angle Architecture of Smart Canopy 1* (*LAC1/DWARF4*) gene encoding a cytochrome P450, CYP90B1, involved in brassinosteroid biosynthesis	*Zea mays* L.	Plants exhibit progressively more erect leaf angles from lower to upper leaves, increased fraction of penetrated PAR and net photosynthesis, increased grain yield per ha at high planting density	Tian et al. (2024)

### Improved light distribution across the canopy

**Figure 2:**
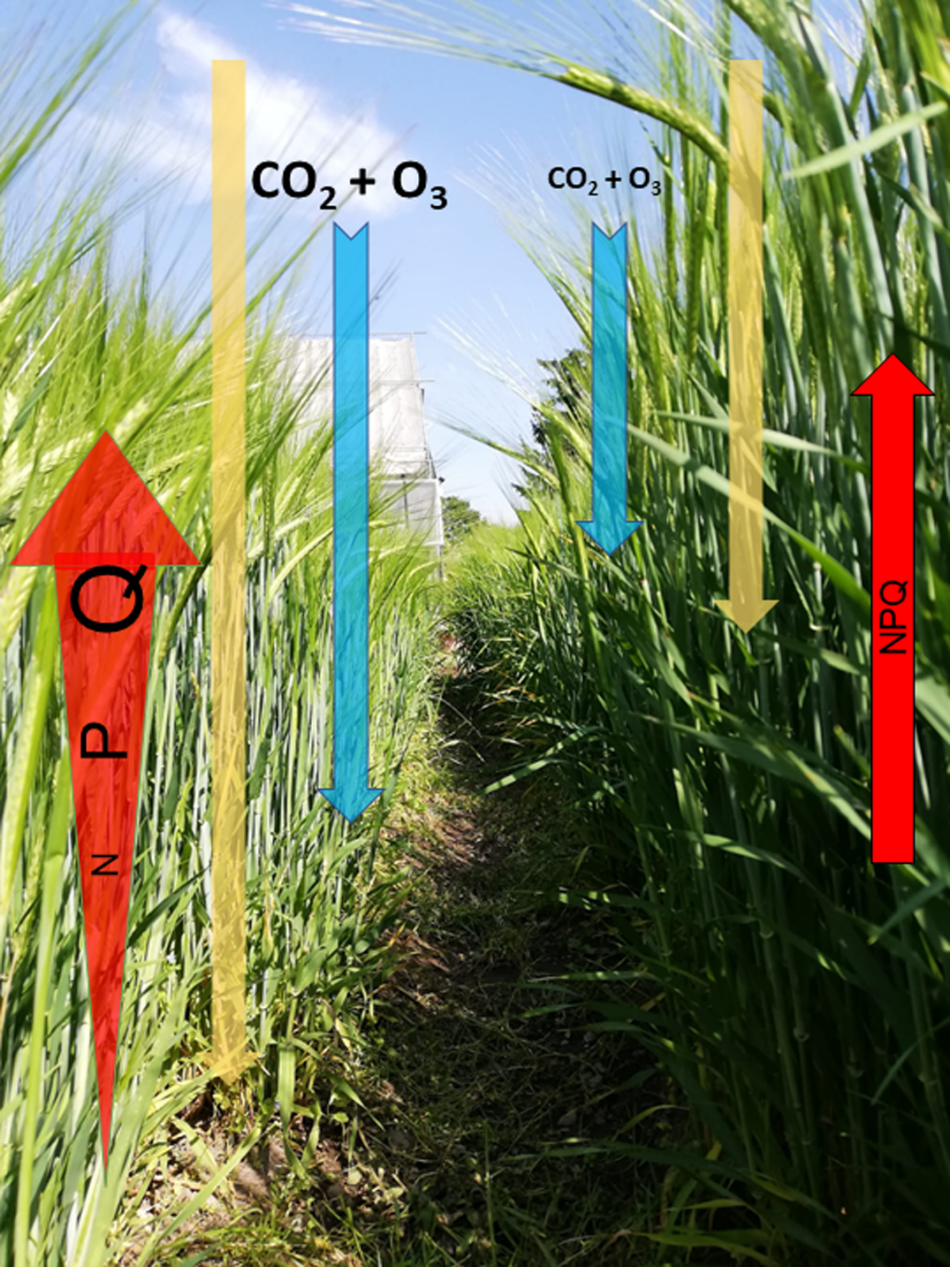
Phenotypic characteristics expected to be observed in advanced breeding lines upon implementation of BEST‐CROP experimental plan. Pale green barley lines (left side), combined with erect leaves will allow a better light penetration through the canopy (yellow arrow), with respect to dark green control plants (right side). These features will be stacked with faster adaptation to rapid light intensity changes (NPQ, red arrow), enhanced gas exchange, and photorespiratory bypasses, allowing an increased rate of CO_2_ fixation and higher O_3_ assimilation (light blue arrow). Image is courtesy of BEST‐CROP partner, UMIL.

Light within a canopy is not absorbed homogeneously, with the leaves more exposed to light absorbing a large fraction, leaving the leaves underneath under light limitation. The reduction of leaf chlorophyll content has been demonstrated to be highly effective in improving light penetration in high‐density mass canopy (Figure 2). Pale green crops can be created by manipulating a plethora of processes, such as the biogenesis and/or accumulation of antenna proteins (Light harvesting complex, Lhc)—also known as the Truncated Light‐harvesting Antenna (TLA) strategy—and pigment biosynthesis (for a review, see Cutolo et al., 2023; Table 1). For instance, increased photosynthetic performance and enhanced plant biomass accumulation were observed upon cultivation at high density under greenhouse conditions of a pale green tobacco line with downregulated expression of *cpSRP43* encoding the 43‐kDa chloroplast Signal Recognition Particle (cpSRP43; Kirst et al., 2018; see also Table 1). cpSRP43 is a chaperone required for post‐translational targeting of Lhc to the thylakoid membranes and contributes to the biogenesis and maintenance of the thylakoid membranes and their associated electron transport chains (Klimyuk et al., 1999; Schuenemann et al., 1998). The corresponding *A. thaliana* mutant is known as *chaos* [chlorophyll a/b binding protein harvesting‐organelle specific (Klimyuk et al., 1999)]. In the chloroplast stroma, cpSRP43 interacts with 54‐KDa chloroplast Signal Recognition Particle (cpSRP54) to form the heterodimeric complex cpSRP. Lhc precursors imported into the stroma from the cytosol are N‐terminally processed and bound by cpSRP to form a soluble cpSRP‐Lhc complex termed the transit complex, which maintains Lhcs in an integration‐competent state (for a review see Ziehe et al., 2018). The transit complex is needed to dock and integrate the Lhc proteins into the thylakoid membranes, with the help of the cpSRP receptor homolog, cpFtsY (Kogata et al., 1999; Tu et al., 1999) and the integral thylakoid membrane protein Albino3 (Alb3), a translocase that physically interacts with cpSRP43 (Moore et al., 2000). Recently, cpSRP43 was also shown to efficiently chaperone and stabilize the glutamyl‐tRNA reductase (GluTR), a rate‐limiting enzyme in tetrapyrrole biosynthesis, enabling Lhc thylakoid insertion to be coordinated with Chl biosynthesis (Wang, Liang, et al., 2018). More recently, the barley mutant *happy under the sun 1* (*hus1*), which carries a premature stop codon in the corresponding *HvcpSRP43* gene and is characterized by a 50% reduction in the chlorophyll content of leaves, was shown to accumulate biomass and grains at levels comparable to those observed for the control cultivar Sebastian, when grown under field conditions at standard density (Rotasperti et al., 2022). These findings demonstrate that crops can indeed decrease their investment in antenna proteins and chlorophyll biosynthesis significantly, without detrimental effects on productivity (Table 1). In barley, a rich variety of mutants exists with a reduced amount of chlorophyll (Hansson et al., 2024). BEST‐CROP will exploit the slight and variable reductions of leaf chlorophyll content observed in *chlorina* mutants. These are light green viable mutants, which can be kept in homozygous stocks. More severe mutants, such as yellow *xantha* mutants, white *albina* mutants, and light green *viridis* mutants, are lethal and not directly suitable for plant breeding. Still, they can be used to identify important genes to be explored for development of plants with a reduced leaf chlorophyll content.

In the “smart canopy” model, reduced chlorophyll content is combined with an optimized plant architecture comprising more erect leaf angles to improve the distribution of photosynthetically active radiation within the crop canopy, increase photosynthetic efficiency and reduce competition among neighboring plants (Ort et al., 2015); these features are designed to allow higher planting densities and increase grain yield (Figure 2). For example, the selection of maize (*Zea mays* L.) hybrids with upright leaves contributed to reaching current densities of over 80 000 plants per hectare in the US Corn Belt compared to 30 000 plants per hectare in the 1930s, which largely account for yield increases in the past decades (Assefa et al., 2018; Duvick, 2005; Tian et al., 2011; see Mantilla‐Perez & Salas Fernandez, 2017 for a review). In grass leaves, the sheath wraps around the stem while the leaf blade or lamina projects away from the stem to facilitate light capture: the leaf insertion angle is defined as the angle between the blade and the vertical stem axis and is regulated by multiple genetic and hormonal factors with brassinosteroids playing a major role. Breakthrough studies demonstrated that specific mutations in orthologous brassinosteroid biosynthesis genes *DWARF4* in rice (*Oryza sativa* L.) and *Leaf Angle Architecture of Smart Canopy 1* (*LAC1*) in maize result in reduced leaf angles and increased grain productivity under high‐density planting (Sakamoto et al., 2006; Tian et al., 2024). In particular, a detailed analysis of the maize *lac1* mutant under field conditions showed that optimized reduction of leaf angles at different canopy levels leads to increased penetration of sunlight to the lower canopy and improved photosynthesis (Tian et al., 2024). A similar result was obtained in sugarcane (*Saccharum* spp. hybrid) where the fine‐tuning of leaf angle through genome editing of the *LIGULELESS 1* gene (*LG1*) has optimized light capture and increased dry biomass yield up to 18% without having to add more fertilizer (Brant et al., 2024). In barley, plants with a reduced leaf angle and an overall erect architecture are especially found among the *Brachytic*, *Erectoides*, and *Breviaristatum* mutant groups (Hansson et al., 2024; see also Table 1).

### Faster responses to changes in irradiance

Under full sunlight, plants dissipate the excess of potentially damaging absorbed light energy by inducing the photo‐protecting mechanism termed nonphotochemical quenching (NPQ). This process is essential to avoid the formation of reactive oxygen species (ROS) that would damage the photosynthetic apparatus. However, NPQ activation/deactivation kinetics are too slow to adapt to the rapid fluctuations of light intensity within crop canopies, due to changing cloud cover and leaf movement. Optimized plant response to these dynamics has been shown to improve photosynthetic efficiency and productivity with Violaxanthin DE‐epoxidase (VDE), Zeaxanthin Epoxidase (ZEP), and the PsbS subunit of Photosystem II (PSII) playing major roles. Kromdijk et al. (2016) demonstrated, for instance, that upregulations of *VDE*, *ZEP*, and *PbsS* significantly accelerate the kinetics of photo‐protection induction and relaxation in tobacco, and this resulted in an increased efficiency of CO_2_ assimilation in fluctuating light conditions, which translated into a 14–21% increase in shoot biomass production in replicated field trials (Figure 2; Table 1). More recently, the same change in an elite soybean (*Glycine max* L. Merr.) variety increased seed yield by up to 33% in small‐scale field trials (de Souza et al., 2022), pointing to NPQ as a novel potential target for increasing photosynthesis efficiency and crop yield. However, these results were not replicated in other species such as Arabidopsis (Garcia‐Molina & Leister, 2020) and potato (*Solanum tuberosum*; Lehretz et al., 2022), indicating that the complexity of light environmental conditions requires tailored and species‐specific optimization of the response. BEST‐CROP will attempt to ameliorate NPQ kinetics by exploiting barley natural genetic variability collected at the Cranachan database (https://barley.hutton.ac.uk/), as well as barley mutant populations available at the James Hutton Institute (Caldwell et al., 2004; Schreiber et al., 2019).

An additional strategy to minimize the over‐reduction of photosynthetic electron transporters in response to light dynamics and the consequent generation of ROS evolved in cyanobacteria, algae, non‐vascular plants, and gymnosperms but not in angiosperms. This photoprotective strategy relies on Flavo‐di‐iron proteins (FLVs; Alboresi et al., 2019), which accept electrons downstream of Photosystem I (PSI) to reduce oxygen to water, decreasing the potential formation of ROS. FLV role is particularly important after an abrupt change in illumination intensity, which causes an imbalance between the photosynthetic electron transport, which instantaneously responds to changes in illumination and NADPH consumption that, instead, depends on the metabolic activity and thus requires several minutes to adjust. Consistent with this activity, mutants depleted in FLV show strong photosensitivity when exposed to light fluctuations, as experienced under field conditions.

Recently, FLV proteins have been shown functional when expressed in Arabidopsis (Yamamoto et al., 2016) and rice (Wada et al., 2018), meaning that their activity does not require specific accessory components absent in angiosperms. Moreover, FLV expression in Arabidopsis (Tula et al., 2020) and tobacco (Vicino et al., 2021) was shown to induce a higher tolerance to abiotic stresses, even though the impact on yield under field conditions was never fully assessed (Table 1). BEST‐CROP aims at expressing heterologous FLVs in barley and verifying whether this strategy could improve crop productivity under natural light fluctuations, especially in conditions such as drought stress, where CO_2_ fixation is limited, and the availability of additional electron acceptors could be beneficial.

### Carbon‐positive photorespiration bypass routes

To develop a carbon‐positive photorespiration bypass pathway, enzymes that do not occur naturally in barley are introduced through *Agrobacterium*‐mediated transformation. This will alleviate the impact of the double activity of RuBisCo that not only catalyzes the carboxylation of ribulose 1,5‐bisphosphate but also its oxygenation (for a review see Smith et al., 2023). The oxygenation reaction reduces the photosynthetic efficiency and, thus, the plant's yield. The oxygenation activity of RuBisCo produces 2‐phosphoglycolate (2PG), which must be recycled by photorespiration. This results in a yield loss of 20–50% of the previously assimilated carbon. BEST‐CROP aims to reduce the yield loss due to photorespiration by 50% by using alternative bypass pathways for photorespiration. Independent research groups have shown that such bypasses can significantly increase yield under laboratory and field conditions in various crops and model plants and offer a promising solution to this challenge (Kebeish et al., 2007; Maier et al., 2012; Shen et al., 2019; South et al., 2019; see also Table 1).

**Figure 3:**
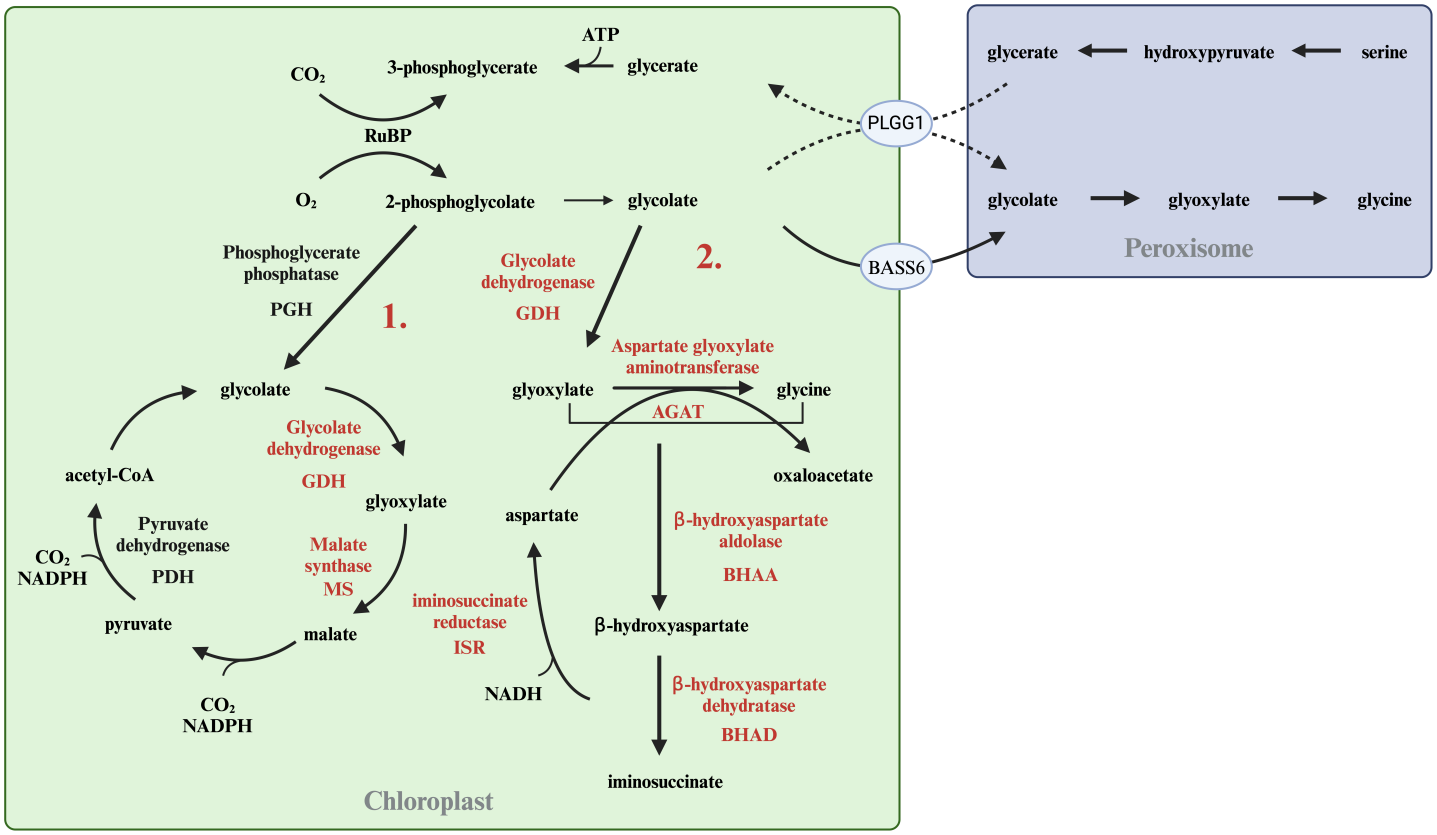
Summary of the two alternative bypasses of photorespiration exploited in BEST‐CROP. (1) In the first bypass, the complete oxidation of 2‐phosphoglycolate to CO_2_ will be obtained through the sequential action of phosphoglycerate phosphatase (PGH) native to barley chloroplasts, glycolate dehydrogenase (GDH) from *Chlamydomonas reinhardtii* mitochondria, malate synthase (MS) from *Cucurbita maxima* (pumpkin) peroxisomes, endogenous NADP malic enzyme, and finally endogenous pyruvate dehydrogenase (PDH). In addition, RNAi‐mediated reduction (indicated by dotted lines) of the plastid glycolate glycerate translocator 1 (PLGG1) reduces glycolate export from the plastids. (2) The second bypass will allow to achieve carbon‐neutral conversion of glycolate to oxaloacetate through a plastid‐targeted version of the beta‐hydroxyaspartate shunt (BHAC). This includes glycolate dehydrogenase (GDH) from *C. reinhardtii* mitochondria, aspartate:glyoxylate aminotransferase (AGAT), β‐hydroxyaspartate aldolase (BHAA), β‐hydroxyaspartate dehydratase (BHAD), and iminosuccinate reductase (ISR). BASS6, bile acid sodium symporter. Endogenous genes are in black, while heterologous genes are in red. Image created in https://BioRender.com.

Two alternative bypasses of photorespiration are exploited in BEST‐CROP (Figure 3). First, the complete oxidation of 2‐phosphoglycolate to CO_2_ by the sequential action of phosphoglycerate phosphatase native to barley chloroplasts, glycolate dehydrogenase from *Chlamydomonas reinhardtii* mitochondria, malate synthase from *Cucurbita maxima* Duch. (pumpkin) peroxisomes, endogenous NADP malic enzyme, and finally endogenous pyruvate dehydrogenase. In addition, RNAi‐mediated reduction of the Plastidal glycolate glycerate translocator 1 (PLGG1) reduces glycolate export from the plastids. Variations of this strategy have been successfully used in several crops and model plants and are considered a low‐risk baseline option (Kebeish et al., 2007; Maier et al., 2012; Shen et al., 2019; South et al., 2019). Second, we will achieve carbon‐neutral conversion of glycolate to oxaloacetate through a plastid‐targeted version of the beta‐hydroxyaspartate shunt (BHAC). This includes glycolate dehydrogenase from *C. reinhardtii* mitochondria, aspartate:glyoxylate aminotransferase, β‐hydroxyaspartate aldolase, β‐hydroxyaspartate dehydratase, and iminosuccinate reductase. This pathway was developed as part of the EU H2020 GAIN4CROPS project, and proof‐of‐concept for a peroxisome‐targeted version in Arabidopsis was provided (Roell et al., 2021). Unpublished work suggests a higher efficiency of a plastid‐targeted beta‐hydroxyaspartate shunt. Based on this, we will introduce a plastid‐targeted version in barley by replacing the peroxisome‐targeting signals with plastid transit peptides. This strategy carries a higher risk with a potentially high gain and complements the lower‐risk bypass described above.

### Excluding stomatal limitations for CO_2_ and O_3_ uptake

**Figure 4:**
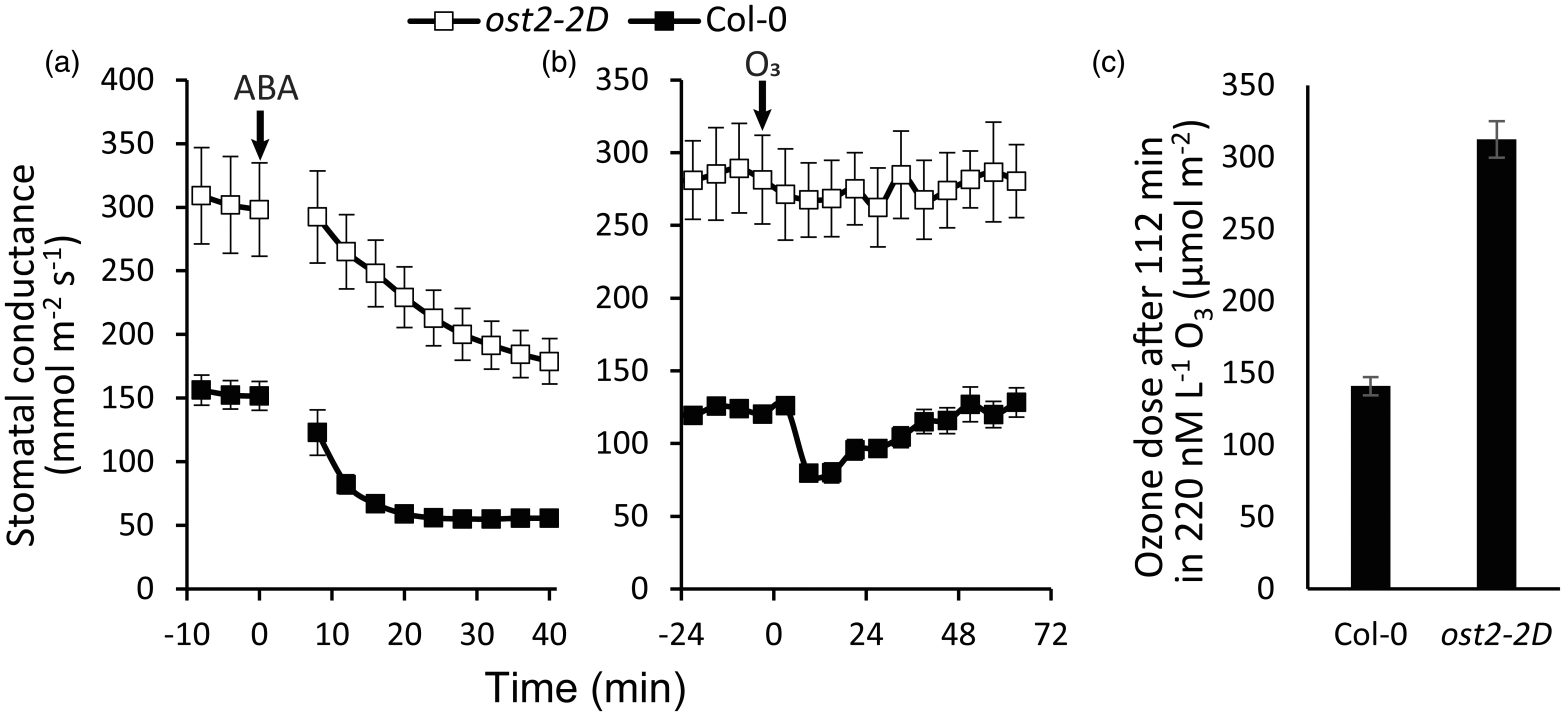
The gain‐of‐function mutation in the proton pump *At*AHA1 increases stomatal conductance and ozone (O_3_) uptake but does not affect the stomatal responsiveness to abscisic acid (ABA) in Arabidopsis. The 3 to 4 weeks‐old plants were incubated in the gas exchange device (Kollist et al., 2007) and were subjected to spraying with 5 μM ABA (a) or 3‐min 450 nM L^−1^ ozone pulse (b). The *ost2‐2D* mutant (Merlot et al., 2007) was compared with wild type plants (Col‐0). The starts of the treatments are shown by the arrows. In another experiment, the Col‐0 and *ost2‐2D* plants were exposed to continuous 220 nM L^−1^ ozone. The cumulative dose of absorbed ozone is shown (c). The values are averages ± SE (*n* = 3–4).

Stomatal pores formed by the guard cells balance plant water loss by evapotranspiration with CO_2_ uptake for photosynthetic carbon assimilation. Vegetation also absorbs significant amounts of air pollutants, such as ozone, mainly through stomatal pores (Brosché et al., 2010). The central role of stomata in controlling plant gas exchange processes makes them an attractive target to enhance photosynthetic efficiency and drought tolerance as well as to manage ozone uptake in crops. Several strategies employing stomata for improving crop productivity have been suggested; these include altering stomatal density and size (Caine et al., 2023; Hughes et al., 2017), accelerating stomatal movements (McAusland et al., 2016; Papanatsiou et al., 2019), and increasing stomatal conductance for elevated CO_2_ uptake by overexpressing proton pumps (Zhang et al., 2021; Table 1). Enhanced stomatal conductance will also ensure improved ozone uptake, although it is important to maintain stomatal responsiveness to stress factors, such as drought, in these plants. Such a strategy could be instrumental in developing crops that help to improve the air quality in regions with a high level of air pollution (Diener & Mudu, 2021; Sicard et al., 2018). In BEST‐CROP, we aim to develop barley lines with moderately increased stomatal conductance, to improve CO_2_ and ozone uptake while maintaining stomatal responsiveness to drought conditions. We will target barley proton pumps providing energy for stomatal opening; we will either overexpress proton pumps or introduce mutations that disrupt their autoinhibition mechanism. Such barley lines will have enhanced stomatal conductance ensuring maximal supply of CO_2_ for photosynthesis and elevated ozone uptake by mesophyll (Kollist et al., 2000). At the same time, these lines will maintain an intact stomatal closure in response to drought stress hormone, abscisic acid (ABA), as demonstrated in Arabidopsis (Figure 4). Alternatively, lines with enhanced stomatal conductance but unaffected ABA/drought responses can be obtained by modulating activities of the proteins involved in CO_2_ sensing in guard cells (Takahashi et al., 2023; Yeh et al., 2023).

To achieve this, we will identify gain‐of‐function mutants for *HIGH LEAF TEMPERATURE 1* (*HT1*; Hõrak et al., 2016) and knockouts for the barley *MITOGEN‐ACTIVATED PROTEIN KINASE 12* homolog (*MPK12*; Tõldsepp et al., 2018) in mutant collections. Stomatal traits in these novel barley lines will be thoroughly characterized by using the custom‐made whole plant gas exchange devices (Hõrak et al., 2017; PlantInvent Ltd).

Cumulative uptake of ozone and its possible toxicity will be estimated upon exposure to ozone concentrations observed in Europe's air‐polluted regions, together with measurements of photosynthesis and stomatal behavior in various environmental conditions. As a potential remedy to mitigate ozone toxicity, overexpression of enzymes involved in scavenging reactive oxygen species, such as superoxide dismutase, can be applied (van Camp et al., 1994).

## IMPROVING PHOTOSYNTHESIS BY EXPLOITING INDUCED MUTANTS IN COMBINATION WITH GENE EDITING AND GENETIC ENGINEERING

Currently, most biotechnological approaches to improve photosynthetic performance rely on mutants as well as on gene editing and genetic engineering approaches. This is possible due to the knowledge gained in more than three decades of functional genomics studies on photosynthesis using model organisms in combination with the ability to model photosynthetic systems to identify critical proteins and enzymes that control photosynthetic efficiency. For instance, the improvement of canopy photosynthesis via the faster recovery of NPQ was predicted, initially, through a dynamic system model of canopy photosynthesis (Zhu et al., 2004), and later validated in both soybean and tobacco (de Souza et al., 2022; Kromdijk et al., 2016). Similarly, modeling of photorespiratory bypass suggested that decreased expression of *PLGG1*, a glycolate/glycerate transporter (Pick et al., 2013), could result in an increased benefit of photorespiratory bypass and further increased photosynthetic CO_2_ uptake rate (Xin et al., 2015), which was later experimentally confirmed in tobacco (South et al., 2019) and rice (Shen et al., 2019). Exploiting intraspecific natural genetic diversity to improve photosynthesis is, instead, still in its infancy. Limitations are imposed by the minor variation in the basic physiological and biochemical mechanisms of the photosynthetic engine within species, as shown by the fact that the key components of photosynthesis [i.e., the subunits of PSII, PSI, and the Calvin‐Benson‐Bassham cycle (CBB)] are highly conserved. Higher rates of photosynthesis have been shown, indeed, to correlate with higher amounts of Cyt *b*_*6*_ *f* complex or RuBisCo per unit area, and not by the identification of Cyt *b*_*6*_ *f* or RuBisCo with higher activity (Miller et al., 2017). Similarly, the accelerated kinetics of NPQ and the improved efficiency of CBB have been obtained by overexpression approaches aimed at increasing the accumulation of specific enzymes in several species, including Arabidopsis, tobacco, rice, soybean, and wheat (for a review see Croce et al., 2024).

On the contrary, a wide diversity of photosynthetic properties and the main components of the photosynthetic apparatus is found in nature across species, and examples of increased photosynthetic carbon assimilation and biomass production in model plants and crops, obtained by overexpression of genes from other species, such as the bifunctional cyanobacterial *sedoheptulose‐1,7‐biphosphatase* (*SBPase*)/*fructose‐1,6‐biphosphatase* (*FBPase*) and algal *Cyt c*_*6*_ (López‐Calcagno et al., 2020), as well as *FLVs* (Tula et al., 2020; Vicino et al., 2021; Wada et al., 2018; Yamamoto et al., 2016), have been reported. This evidence, together with the improved photosynthetic performance obtained through engineered synthetic pathways to bypass photorespiration (for a review see Smith et al., 2023), highlight the relevance of mutants, as well as of gene editing and genetic engineering approaches for increasing crop production. In particular, the gene editing technology, namely CRISPR/Cas, is still evolving, with new enhancements in editing precision, multiplex editing, and gain‐of‐function strategies, although challenges remain in optimizing CRISPR delivery methods and transformation efficiency (Ahmar et al., 2024). Recently, the European Union has taken steps toward embracing New Genomic techniques (NGTs), with a new regulation passing its first vote in the European Parliament (Katsarova, 2024), although NGTs will still have to go through several approvals before they can be used for crop breeding also in Europe.

Currently, the adoption of these different biotechnological strategies led to increases in biomass accumulation ranging from 10 to 68%, although most of the studies were conducted in greenhouses or small‐scale field trials (Khaipho‐Burch et al., 2023). Moreover, no data are available on the adoption of these approaches in different combinations, albeit it is anticipated that altering crucial pathways in plant metabolism, including light conversion and carbon fixation, will inherently have interdependent effects that require additional investigation, through predictive mathematical models and experimental validation of predictions. Noteworthy, even a modest improvement of photosynthesis can lead to a marked increase in productivity according to the Blackman model (Blackman, 1919).

BEST‐CROP is aimed to fill this gap, by combining these strategies into barley elite germplasms and by evaluating their performance under field conditions, taking care to adhere to well‐established testing methods. This includes standard definitions of yields, obtained through field trials replicated across plots, geographical locations, and years, where agriculture practices closely match the conditions of the farms that will ultimately produce the crop (Khaipho‐Burch et al., 2023). It is reasonable to expect that under highly dynamic environmental conditions, in the presence of environmental stressors and pathogens, the increase in productivity of the novel barley lines will be less than the ones observed under controlled conditions and closer to what breeders consider true breakthroughs in crop productivity, that is, yield increases of the order of 5–10%, although combined improvements could potentially yield an additive outcome, maximizing the overall impact on productivity.

## IMPROVING PHOTOSYNTHESIS IN CONNECTION WITH THE OPTIMIZATION OF STRAW QUALITY FOR THE CIRCULAR BIOECONOMY

In the last decade, several research programs have investigated the possibility of valorizing annual plants in various industrial sectors. The BFF project (Biomass For the Future; https://anr.fr/ProjetIA‐11‐BTBR‐0006), for instance, aimed to optimize miscanthus (*Miscanthus* Andersson) and *Sorghum* production and develop new end‐uses of these biomasses for energy (anaerobic digestion) and materials (polymer composites for automotive and construction materials). AGRIMAX (Multiple high‐value products from crop and food‐processing waste; https://agrimax‐project.eu), instead, had the objective to develop and demonstrate the production of multiple, high‐value products (packaging, food additives, fertilizers) from various crops and food‐processing wastes. In addition, GRACE (GRowing Advanced industrial Crops on marginal lands for biorEfineries; https://www.grace‐bbi.eu/) had the ambition to optimize various value chains for the bio‐based industry from miscanthus and hemp (*Cannabis sativa* L.) grown on marginal lands, that is, polluted or non‐arable. Despite stimulating and interesting outcomes, existing major crops such as barley (60 million ha of world arable land), and wheat (240 million ha in the world) were not at the center of these projects, which seems critical given the huge amount of residual straw involved. On the other hand, research projects investigating novel biotechnological strategies able to increase crop photosynthesis performance and total biomass production and grain yield (see also recent EU‐funded projects such as CAPITALISE, GAIN4CROPS, and PhotoBoost) did not take into consideration the need to tailor the quality of the residual straw to be transformed into new bio‐based products.

In BEST‐CROP, the optimization of canopy photosynthesis performance will be coupled with the improvement of barley straw quality, closing the loop on resource use, and waste generation in agro‐ecological systems. While cereal straw is a relevant postharvest by‐product that can be used as an ideal ground cover to prevent soil erosion or incorporated into the soil to improve soil organic matter, it is estimated that, at a grain yield >6 ton/ha, around 60% of barley straw can be removed from soil without depleting soil organic carbon (Johnson et al., 2006). In addition, straw removal from fields prevents nitrogen sequestration by the decomposing straw (Fontaine et al., 2020), further supporting the use of straw for developing high‐value bio‐based materials and compounds.

### Barley straw with different cellulose/lignin contents and lignin properties for the construction and biocomposite sectors

Over the last two decades, there has been a widespread global campaign toward the development and utilization of more sustainable manufacturing materials obtained from plant fibers to ensure a successful transition from a linear economy model to a circular, sustainable bioeconomy (Alaneme et al., 2023; Butu et al., 2020).

Currently, the use of non‐wood plant fibers as supplements or as direct substitutes for wood in particleboards and as reinforcements in biocomposites, that is, a broad class of materials in which natural fibers, used as reinforcement, are combined with a petroleum‐ or bio‐based matrix to form a composite material with superior properties, have been identified as sustainable alternatives to conventional synthetic materials because of their competitive properties and environmentally friendly characteristics (Ahmad et al., 2022). In the case of agricultural residues (e.g., cereal straws, bagasse) for instance, their use should allow reduced consumption of raw materials from petroleum and/or forestry resources and are rapidly renewable.

**Figure 5:**
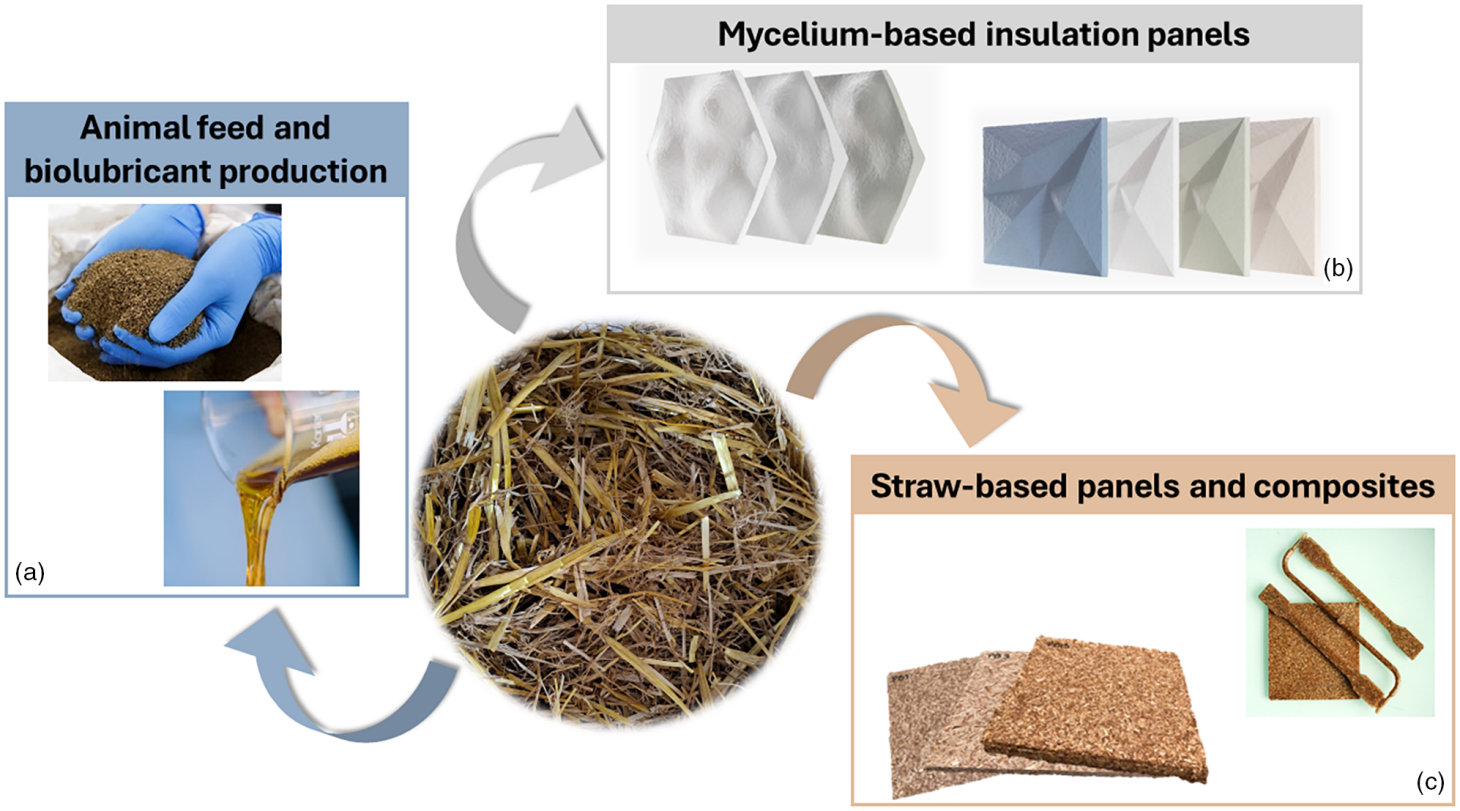
Examples of high‐value bio‐based industrial products that can be obtained from the transformation of barley straw. (a) The use of BSFL (black soldier fly larvae) can transform barley straw into high‐quality protein and fat‐rich biomass suitable for animal feed and biolubricant production; (b) Straw with various lignin content and properties can be used as raw material for manufacturing mycelium‐based insulation panels, and (c) straw‐based panels and composites. Images are courtesy of BEST‐CROP partners: FRD‐CODEM and IMT Mines Alès (straw‐based panels and composites); MOGU srl (mycelium‐based insulation panels); SO.G.I.S. Industria Chimica SpA (animal feed and biolubricants); S.I.S. Società Italiana Sementi (barley straw).

**Figure 6:**
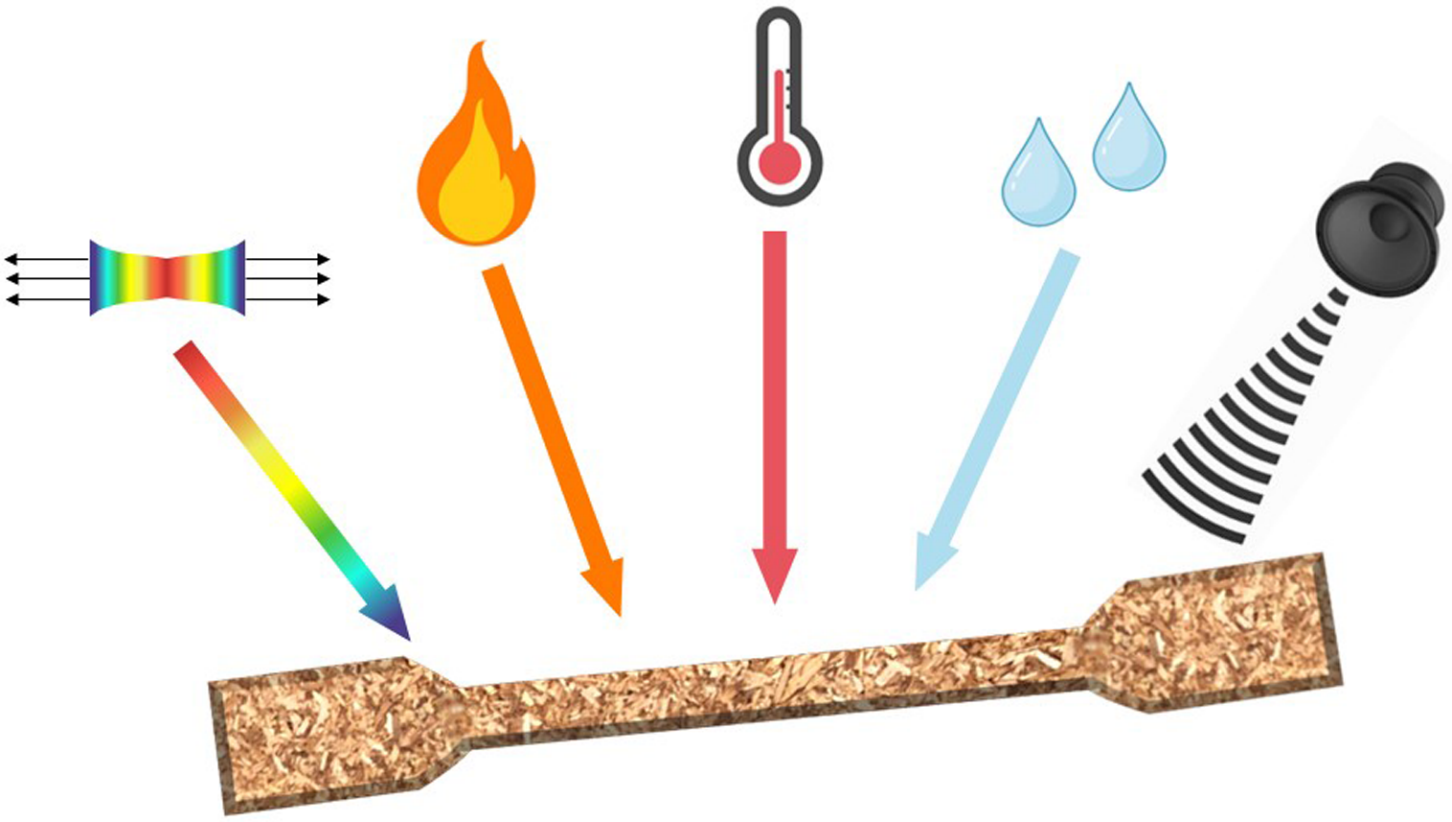
Physical properties of straw‐based materials. Mechanical, flammability, thermal and acoustic insulation, and hygroscopicity properties of mycelium‐based insulation panels and straw‐based panels and composites to be studied in BEST‐CROP in relation to the different barley cultivars selected and the intrinsic characteristics of the cell walls.

Some examples of plant‐based particleboards and biocomposites are already available in the building and construction sector, food packaging, sports, and leisure and automotive industries, driven by the major contribution of these sectors to environmental issues (Figure 5). These industries are, indeed, responsible for a large percentage of carbon emissions and the generation of non‐biodegradable waste, which in turn has adverse effects on ecosystems (Haneef et al., 2017; Peñaloza, 2017; Siddique et al., 2016; Yang et al., 2021). Despite the many factors favoring the use of plant‐based particleboards and biocomposites, there is still some way to go before they are considered reliable alternatives to conventional materials in terms of market uptake. Indeed, their industrial use requires reproducibility and consistency in the morphological and mechanical properties of plant fibers, which are influenced by various cell wall characteristics, including the concentration of cellulose and non‐cellulosic components (i.e., lignin, hemicelluloses, pectins, proteins), microstructure (microfibrillar angle, MFA of cellulose microfibrils), and hydrogen bonds and crosslinks between cell wall components (Bourmaud et al., 2018; Mohanty et al., 2018). One reason for apparent lack of consistency is that plant cell walls and fibers from different crops (and to a lesser extent from different cultivars of the same crop) can have wide diversity in morphology, composition, and mechanical properties. This relates to their differing function within plants, for example the soft seed fibers of cotton help the seeds to disperse and are made of pure cellulose, while stem structural fibers can be flexible with low lignin content (e.g., the phloem bast fibers of crops like flax, hemp, and ramie) or inflexible with relatively high lignin for example in the straw of cereal crops or in wood. Little research has been performed to directly link cell wall properties in a given species and cultivar with the performance of biocomposites produced from that plant material. This means that it is currently not possible to infer, except in the most general terms, how a plant material will perform or how it might be improved for specific biocomposite applications. Better understanding of the relationships between physical properties of barley straw‐based materials and cell wall properties is thus necessary and this is precisely what is proposed in BEST‐CROP to inform the use of barley straw in different applications (Figures 5 and 6).

Flax is a crop currently being used in biocomposites due to its long bast fibers with particularly low lignin and high cellulose content. At the scale of flax elementary fiber cells or fiber bundles, the mechanical properties result from the complex biomolecular assemblies of cellulosic and non‐cellulosic components and their organization within the cell walls. In particular, it has been shown by the selective removal of non‐cellulosic components from flax fibers that these play a key role in the mechanical behavior of the fibers (Lefeuvre et al., 2015). The authors assumed that non‐cellulosic components, that is, matrix and structuring polysaccharides, contribute to the cohesion of cellulose microfibrils and to load transfer within cell walls, which can greatly affect the tensile stiffness and strength of the fibers.

Variations in stiffness can occur even between different genotypes of the same crop, for example in a study of stem fragments from six different miscanthus genotypes with contrasting biochemical compositions, variations in stiffness were related to their biochemical composition, that is, lignin, hemicelluloses, but also *p*‐coumaric and ferulic acids that are proposed to play a role in both cross‐linking between cell wall components and inter‐cellular cohesion (Chupin et al., 2020).

At the composite scale, using stem fragments from different miscanthus and sorghum genotypes with varying biochemical and microstructural features of the cell walls was also found to have a significant influence on the mechanical performance of composite materials. In particular, improvements in the tensile stiffness and strength of miscanthus‐reinforced composites was shown to be correlated with increases in cellulose, lignin, and *p*‐coumaric acid content (Brancourt‐Hulmel et al., 2021; Chupin et al., 2017; Di Giuseppe et al., 2021). Variations in biochemical composition and histological organization among genotypes are also responsible for different breakage mechanisms of stem fragments during composite processing, leading to variations in their mechanical properties related to their microstructure, and the distribution of fiber size and shape within the composites (Vo et al., 2017). Moreover, it has been shown for miscanthus‐based concrete that sugar‐based molecules, in particular xylose and glucose, can hinder the cement hydration process due to their release into the cement–water–sand mixture and their adsorption onto cement particles, which is detrimental to the resulting mechanical strength of concrete blocks (Boix et al., 2020). These studies on plant fibers and their use as reinforcements in composite and concrete materials highlight the need to better identify the key biological traits and related cell wall characteristics that should be optimized for better selection of high‐performance plant fibers and genotypes for material and structural applications.

Flammability of lignocellulosic fibers or stem fragments is also strongly related to their biochemical composition, in particular to the quantities and nature of polysaccharides and lignin, although their fire properties cannot be easily deduced due to the existing interactions between these components (Dorez et al., 2014). Lignin is a high‐charring polyphenol that decomposes slowly over a wide temperature range, while cellulose decomposes more rapidly with a peak mass loss centered at around 360°C and leaving only a small residue. When present in significant amounts and depending on their chemical structure, extractives can also influence the flammability. Therefore, the energy released in case of combustion may vary to some extent. Moreover, the above‐mentioned parameters influence the fire behavior of composite materials incorporating these bioresources. For example, in bio‐based concretes, in which stem fragments are the only organic components likely to burn, the risk of ignition greatly varies depending on the cell wall characteristics of the bioresource used (Lopes et al., 2024).

BEST‐CROP addresses this challenge by modulating lignin/cellulose content and lignin properties to determine the most advantageous composition for efficient implementation of barley straw in high‐value bio‐based industrial products for the construction, and composites sectors. Milled barley straws in the form of fibers, granulates, and flour, will be used as substrates for inoculation and colonization by fungal strains, and the manufacture of straw‐derived mycelium‐based construction panels targeting thermal and acoustic insulation for wall, floor, and ceiling tiles (Alaneme et al., 2023; Attias et al., 2020; see also Figure 6). BEST‐CROP will also consider the valorization of barley straw for the manufacture of straw‐based particle boards with the use of bio‐based binders with enhanced thermal insulation and mechanical properties (Amziane & Collet, 2017; Li et al., 2023; Mahieu et al., 2021; Nakanishi et al., 2018; Tlaiji et al., 2022; Uemura Silva et al., 2021), as well as the use of straw as reinforcements in thermoplastic processes for the manufacture of polymer composites (Bourmaud et al., 2018; Mohanty et al., 2018). To this end, BEST‐CROP will exploit the natural variability in straw lignin content observed to vary from 18.96 to 27.8% when hundreds of elite spring barleys were phenotyped (Halpin et al., unpublished). Moreover, prior work demonstrates how lignin content can be reduced by suppressing lignin biosynthesis genes (Table 2). For instance, a range of transgenic barley plants have been produced where most genes on the lignin biosynthesis pathway have been individually suppressed or knocked out using RNAi or CRISPR‐Cas9 mutagenesis (e.g., Daly et al., 2019; Shafiei et al., 2023). Genes manipulated include *phenylalanine ammonia‐lyase* (*PAL*), *coumarate 3‐hydroxylase* (*C3H*), *caffeic acid 3‐O‐methyltransferase* (*COMT*), *4‐coumarate‐CoA ligase* (*4CL*), *cinnamoyl‐CoA reductase* (*CCR*), *ferulate 5‐hydroxylase* (*F5H*), and *cinnamyl alcohol dehydrogenase* (*CAD*). Lignin composition and structure were dramatically altered in all lines and lignin content was either unchanged or reduced. Downregulation of *F5H* substantially altered the structure of lignin by changing the ratio of syringyl‐to‐guaiacyl monomers in the polymer, but the mechanical properties and yield were unchanged illustrating the plasticity of the polymer and opportunities to tailor it to improve lignocellulosic biomass as a feedstock for green chemistry and specific straw applications (Shafiei et al., 2023). For example, *COMT* RNAi lines, like *F5H* RNAi lines, had dramatically reduced syringyl‐to‐guaiacyl monomer ratio (reduced by 50%), a 15‐fold increase in the amount of 5‐hydroxyguaiacyl units, but also had moderately reduced lignin content (up to 15% reduction) and some improvement in digestibility (Daly et al., 2019). Improved digestibility should mean that sugars can be more easily hydrolysed from the straw biomass as a substrate for growing black soldier fly larvae (see next section) or by mycelium‐generating fungi producing architectural panels. However, defining the best ideotype of barley for industrial purposes in terms of straw composition (cellulose, lignin, and other cell wall components) must await the results of BEST‐CROP after straws of different compositions are tested in various applications. To manipulate lignin even further and, for example, to increase lignin content, different approaches can be exploited. The *chalcone synthase colourless2* (*C2*) gene, controls the flux toward flavonoid biosynthesis in maize vegetative tissues and, when silenced, flux is redirected in lignin monomer synthesis causing strikingly higher lignin content in leaves (Eloy et al., 2017). Similarly, some *brittle culm* (*bc*) mutants of rice with altered cellulose deposition have increased levels of lignin, for example, the *bc1* mutant affected in a *COBRA‐like* gene (Li et al., 2003), *bc10* a mutant in a Domain of Unknown Function 266 (DUF266)‐containing type‐II membrane protein (Zhou et al., 2009), and *bc12* a kinesin‐4 protein (Zhang et al., 2010). Another novel way to manipulate cell wall properties is to alter the amount of ferulic acid involved in cross‐linking between arabinoxylan and lignin, and the amount of *p*‐coumaric acid also bound to arabinoxylan and lignin. A natural mutation that achieves this by knocking out a *p‐coumaroyl acyltransferase* (*PAT*) gene in barley grain has been discovered, recently (Houston et al., 2023).

**Table 2:** Main proof‐of‐concepts, demonstrating that straw quality can be modified

Optimization of barley straw				
Trait	Target gene(s)/germoplasm(s)	Tested Species	Level of accumulation	References
*Straw/stover with different cellulose/lignin contents and lignin properties*				
Reduced lignin	Downregulation or mutation of *4‐coumarate‐CoA ligase* (*4CL*)	*Oryza sativa* L.; *Sorghum bicolor* (L.) Moench	16–20% lignin reduction in both species; 17% increase in sugar yield on saccharification demonstrated in sorghum	Saballos et al. (2008, 2012) and Gui et al. (2011)
	Downregulation or mutation of *cinnamoyl‐CoA reductase* (*CCR*)	*Zea mays* L.; *Lolium perenne* L.	Lignin content reduction by 9–37%, with 14–15% improved digestibility; up to 40% improved glucose yield on saccharification in maize	Tu et al. (2010), Tamasloukht et al. (2011) and Smith et al. (2017)
Increased lignin	Mutation in *chalcone synthase* (*CHS*)	*Z. mays* L.	Increased leaf lignin and reduced leaf saccharification	Eloy et al. (2017)
	Mutation in *brittle culm1* (*bc1*) encoding COBRA‐like protein	*O. sativa* L.	Increased lignin and reduced cellulose in developing sclerenchyma	Li et al. (2003)
	Mutant (*bc10*) in a *Domain of Unknown Function 266* (*DUF266*)‐containing type‐II membrane protein	*O. sativa* L.	Increased lignin and reduced levels of cellulose and arabinogalactan proteins (AGPs)	Zhou et al. (2009)
	Mutation in *brittle Culm12* (*bc12*) encoding kinesin‐4 protein	*O. sativa* L.	Increased lignin with no change in cellulose	Zhang et al. (2010)
Altered lignin structure and composition	Downregulation of *caffeic acid O‐methyltransferase* (*COMT*)	*L. perenne* L.; *Z. mays* L.; *Hordeum vulgare* L.	Generally: Lignin syringyl‐to‐guaicyl ratio reduced; 5‐hydroxyguaiacyl lignin units increased; Improvement in digestibility and/or saccharification	Pichon et al. (2006), Tu et al. (2010) and Daly et al. (2019)
	Downregulation of *ferulate‐5‐hydroxylase* (*F5H*)	*O. sativa* L.; *H. vulgare* L.	Lignin syringyl‐to‐guaicyl ratio reduced by up to 85%	Takeda et al. (2017) and Shafiei et al. (2023)
	Downregulation or mutation of *cinnamyl alcohol dehydrogenase* (*CAD*)	Many grasses for example, *Z. mays* L.; *Festuca arundinacea* Schreb.	Generally reduction in syringyl lignin units and syringyl‐to‐guaicyl ratio; New lignin monomers detected (sinapaldehyde, coniferaldehyde); Digestibility, saccharification, or increased ethanol yield reported in some studies	Halpin et al. (1998), Chen et al. (2003), Fornalé et al. (2012) and Barrière et al. (2013)
Cell wall crosslinks	Downregulation of *feruloyl transferases* (*FAEs*)	*O. sativa* L.	19% reduction in ferulic acid in cell walls	Piston et al. (2010)
	Overexpression of *feruloyl‐CoA monolignol transferase* (*FMT*)	*O. sativa* L.	Increased lignin‐associated ferulate esters	Karlen et al. (2016)
	Mutation in BAHD *p*‐coumaroyl arabinoxylan transferase (*HvAt10*)	*H. vulgare* L.	Reduction in esterified *p*‐coumaric acid and increase in ferulic acid in grain cell walls	Houston et al. (2023)
*Straw with increased protein content*				
Crude protein content	Exploit natural variation for the trait (e.g., 200 spring advanced breeding lines from ICARDA—Morocco)	*H. vulgare* L.	Crude protein content varies between 3 and 6.5% of straw weight	Sanchez‐Garcia et al., unpublished
Nitrogen content	Mutation in *abnormal cytokinin response1 repressor1* (*HvARE1*)	*H. vulgare* L.	Four times more nitrogen content in shoots of *are1* mutant grown under low nitrogen conditions	Karunarathne et al. (2022)
Nitrogen use efficiency	Mutation in *Bric‐a‐Brac/Tramtrack/Broad gene* (*OsBT2*)	*O. sativa*	Mutations of this gene in rice increased NUE by 20% compared with wild type under low N conditions	Araus et al. (2016)
Nitrogen translocation	Mutation in *Lysine‐Histidine‐type Transporter 1* (*OsLHT1*)	*O. sativa*	At maturity, only 40% of total N was translocated to reproductive tissues in mutants (62% in wild type plants)	Guo et al. (2020)

### Barley straw with increased protein amount for the feed and green chemistry sectors

Low‐grade organic waste, as in the case of cereal straws, can also be converted at a high rate, by the fast‐growing black soldier fly larvae (BSFL; *Hermetia illucens* L.), into high‐quality protein (41–54% of dry matter) and fat‐rich biomass (11.8–41.7% of dry matter) suitable for animal feeding and biolubricant production, contributing to recycling nutrients from the environment in the frame of the circular bioeconomy (Oonincx & de Boer, 2012; Seyedalmoosavi et al., 2022). The EU Commission has set a target of 20% of chemicals from renewable sources by 2030 (COM 2021/800/EC—Sustainable Carbon Cycles), and oleochemistry is the most appropriate technology to foster the transition from mineral oil chemistry to chemistry from renewables. Palm oil, an essential raw material for oleochemicals production, is under issue because of deforestation and, in general, it is considered a non‐sustainable or a scarcely sustainable raw material. Insects can be a useful alternative, and BSFL accumulate large amounts of fat with a prevalence of saturated fatty acids (76% of total fat, including C12 and C16 chains; Renna et al., 2024), making its oil composition similar to that of palm oil. In addition, the aminoacidic composition of BSFL proteins makes them suitable as an alternative protein source with respect to soybean meal and fishmeal. Leucine, lysine, and valine are the most abundant amino acids in BSFL proteins, and they are more abundant than in soybean and fishmeal, while the less abundant essential amino acids, methionine, and tryptophan, are comparable to soybean (Lu et al., 2022). Thanks to its aminoacidic profile, BSFL has been used as a partial replacement for soybean and fishmeal in pig, broiler, and fish diets (Chia et al., 2019; Dabbou et al., 2018; Nogales‐Mérida et al., 2019; Yu et al., 2019).

Larvae of BSF are highly efficient in converting organic waste, including agricultural residues, into biomass. However, residues such as the lignocellulosic cereal straw do not contain enough protein to support BSFL fast growth (Fuso et al., 2021) and enriching them by adding vegetable proteins (e.g., soybean), strongly increases costs. BEST‐CROP aims at developing advanced barley cultivars with increased straw protein content, thus improving its nutritional quality and nutrient balance as a component of the BSFL feeding substrate.

Natural genetic variation for straw protein accumulation remains largely unexplored, with limited available data estimating a 2–6% crude protein content (Keno et al., 2021; Wamatu et al., 2019). Recently, ICARDA barley breeding programs evaluated crude straw protein content as a valuable selection parameter on over 200 breeding lines, confirming a straw protein content ranging from 3 to 6.5%, with a heritability of 0.51 and a weak negative correlation with aboveground biomass (Sanchez‐Garcia et al., unpublished; Table 2). Similarly, little is known about nitrogen partitioning in the different organs of barley plants (Barmeier et al., 2021) and the differences between malting and feeding barley, selected for low/moderate and high grain protein content, respectively, in their ability to mobilize nitrogen from the stem to grains. Genes involved in nitrogen use efficiency (NUE) or protein remobilization from leaves to developing grains, can be identified in close crop species from literature data, and they can be considered as possible targets for improving barley straw protein content. For instance, loss of function mutations in the rice *abnormal cytokinin response1 repressor1* (*OsARE1*) gene have been associated with improved N utilization (Wang, Nian, et al., 2018). Moreover, CRISPR/Cas9‐mediated editing of the barley ortholog *HvARE1* caused delayed senescence and increased content of total chlorophyll in the flag leaf at the grain filling stage. Under low nitrogen conditions, barley *are1* mutants showed high nitrogen content in shoots (Karunarathne et al., 2022). *BT2*, a member of the Bric‐a‐Brac/Tramtrack/Broad gene family, represses the expression of nitrate transporters, thus playing a central role in the NUE network. Mutations of this gene in rice increased NUE by 20% compared with wild type under low N conditions (Araus et al., 2016). The rice *Lysine‐Histidine‐type Transporter 1* (*OsLHT1*) is involved in the translocation of amino acids from vegetative to reproductive organs for grain yield and quality. The concentrations of total N in the flag leaf at maturation were higher in *Oslht1* mutants than wild type rice plants, but a reduction of panicle length, seed setting rate, and total grain weight was also observed (Guo et al., 2020). BEST‐CROP will investigate allelic variation for these genes within available barley germplasm collections and TILLING populations, and will generate novel mutants through CRISPR/Cas9, with the final aim of improving the protein content of barley straw and promoting its use as substrate for BSFL growth.

## PERSPECTIVES ON INNOVATIVE TECHNOLOGIES AND ENVIRONMENTAL AND ECONOMIC BENEFITS

### Impacts from increased barley productivity

By increasing the productivity of barley (both grain and biomass) up to 5–10%, BEST‐CROP could contribute to almost 8 Mt more grain and 5 Mt more removable straw per year if this was replicated globally, potentially contributing to food security. Improving the photosynthetic efficiency of barley will also offer major advantages for the environment, given that around 4% of arable land is used for barley cultivation, globally. Agriculture has, indeed, an active role in the emission of greenhouse gasses to the atmosphere (Kabange et al., 2023). According to the Green House Gases (GHG) inventories of the US Environmental Protection Agency (EPA), GHG emissions in agriculture accounted for almost 22% of total global GHG emissions, a non‐negligible amount (EPA, 2024). The new barley lines developed by BEST‐CROP are expected to convert more CO_2_ to biomass and soil carbon, which will directly contribute to decreasing agricultural contribution to greenhouse gas emissions. The impact of agriculture on climate change and GHG emissions is largely driven by the production of ammonia for agriculture, which currently takes 2–3% of the total world energy (Pfromm, 2017), thus saving nitrogen use is key to reducing energy consumption and GHG release. Further, nitrogen runoff from agricultural land is a major pollutant. Therefore, reducing the use of nitrogen fertilizer will help to minimize this problem. With respect to this issue, the adoption of pale green barley lines in BEST‐CROP is of critical importance since reduced leaf chlorophyll content has been associated with a 9% decrease in nitrogen inputs (Walker et al., 2018). Moreover, photorespiration lowers crop nitrogen use efficiency because ammonia (NH_3_) is released by the glycine decarboxylase reaction in mitochondria during photorespiration. Reducing the rate of photorespiration and/or restricting photorespiratory nitrogen release by introducing photorespiratory bypasses will contribute to the unproductive loss of nitrogen from plants. In this context, it is interesting to note that approximately 90% of the C3‐photosynthesis leaf NH3 assimilation machinery is busy with re‐assimilation of photorespiratory ammonia, while only approximately 10% is involved in net N‐assimilation (Keys, 2006). Pale green leaves in crops will also have a major role in climate change mitigation since they will increase the surface albedo. Modeling experiments have shown that higher cropland albedo may effectively mitigate the magnitude of future heatwaves and global warming in general (Kala et al., 2022) by lowering near‐surface air temperatures (Seneviratne et al., 2018) and transpiration water loss, leading to a 25% reduction of leaf‐level water use (Głowacka et al., 2018). Furthermore, BEST‐CROP aims to increase stomatal O_3_ assimilation without yield penalty. This will mitigate O_3_ spikes and improve air quality. Noteworthy, some of the biotechnological strategies adopted in BEST‐CROP have been already shown to work also in other crops including, among others, wheat, rice, tomato (*Solanum lycopersicum* L.), soybean, and maize, thus cultivation of crops with enhanced photosynthesis performance and ozone uptake is expected to provide a major contribution to the sustainability of agriculture and mitigation of climate change.

### Impacts from barley straw‐based construction panels and composites

Straw with various lignin content and properties can be used as raw material for different manufacturing pipelines. This includes mycelium‐based and straw‐based panels and composites for the construction and composite sectors. In the past 20 years, the production volume of wood‐based panels for building and construction has almost doubled from approximately 180 million m^3^ in 2000 to over 368 million m^3^ in 2020 (FAOSTAT‐Forestry Database, https://www.xresearch.biz/shop/biocomposites‐market). Also, the global biocomposite market is expected to grow from USD 24 726.7 million in 2022 to USD 74 593.5 million in 2030 (XResearch Company, 2023). The market attractiveness for these materials and the actual and projected increase in production volumes offer excellent prospects for the use of other biomasses as alternatives to wood and synthetic materials. In particular, the valorization of residual straws will have a considerable impact on the circular bioeconomy and climate change mitigation. Various lignocellulosic sources and agricultural production processes, including traditional plant fibers (e.g., flax, hemp, jute, sisal kenaf) and agro and forestry residues (e.g., cereal straws, corn stover, grasses such as miscanthus; Mahieu et al., 2019; Mohanty et al., 2018; Neitzel et al., 2023; Pędzik et al., 2021), have shown clear potential as alternative raw materials for the manufacture of particle boards and composite materials with excellent mechanical, insulation and fire properties (Andrew et al., 2024; Bekhta et al., 2023; Bourmaud et al., 2018; Lee et al., 2022; Rosa Latapie et al., 2023). Furthermore, annual production volumes of residual cereal straws are consistent with the huge demand for materials in these sectors. The sustainable solutions exploited in BEST‐CROP will enter the economically relevant building insulation material market that has been valued globally at USD 27.84 billion in 2020, and it is projected to register a CAGR (Compound Annual Growth Rate) of 4.35% during the period 2021–2026.

### Impacts from barley straw‐derived biolubricants and proteins

By gaining oils from BSFL fed with protein‐enriched barley straw, BEST‐CROP will deliver sustainable solutions for the oleochemical industry as well as proteins for feed. This strategic chemical sector is facing significant difficulties in raw material supply due to competition from biodiesel/biofuels and deforestation regulations, and sustainability rules, leading to uncertainties over future availability of palm and soybean oils. Oil from BSFL will offer sustainable and local solutions to substitute plant and fossil‐fuel derived oils, and possibly increase guarantees on supply security. Moreover, several other substances can be obtained from BSLF oil finding application in a variety of industrial sectors, like building industry, cosmetics, pharmaceuticals and animal feeding.

In addition to being biodegradable and non‐toxic, biolubricants have been shown to exhibit superior properties over conventional lubricants (Syahir et al., 2017). In this context, the demand for biolubricants, such as lubrorefrigerant oils and metalworking lubricants, is expected to increase soon (for applications such as hydraulic oils, engine oils, gear oils, cutting fluids, electrical appliances, and turbomachinery) as an alternative to conventional lubricants, especially in uses entailing leakages (Sarma & Vinu, 2022). The global biolubricant market was estimated at 1.9 billion USD in the year 2020 and is projected to reach 2.5 billion USD by 2026, growing at a CAGR of 5.2% over the analysis period (Grand View Research, 2023). Moreover, proteins obtained from BSFL‐based conversion of barley straw will help to cover the existing shortage in local protein production. Being sustainable and free from indirect land‐usage change (ILUC) risk, these proteins could be used to feed fish, non‐ruminant animals and, in the case of hydrolysates, free amino acids can also be used for ruminants.

## CONCLUDING REMARKS

Current environmental and societal issues, together with existing and future policies, are driving industries to develop eco‐efficient, bio‐based compounds, and materials in various sectors, such as chemical industry, building & construction, transportation, and sports & leisure. Besides the market size and growth expectations, the current availability of sustainable raw materials offers great opportunities. To this end, a variety of lignocellulosic sources and agricultural production processes are being considered, including traditional plant fibers and agricultural and forestry residues. The deployment of crop‐straw and plant‐based products will promote the creation of new industrial facilities, offering new growth opportunities, particularly for farm‐based rural areas, where the diversification of economic activities and new industrial outlets are crucial for generating added value in agriculture‐related fields, while contributing to the decarbonization of corresponding industrial sectors. The multi‐purpose barley cultivars delivered by BEST‐CROP will support straw‐based applications and innovative technologies that can be arranged, even on a small scale, in any rural area. This is a huge advantage for the supply chain, allowing the entire production process to be covered in a single compact plant—from raw material to final product. Finally, BEST‐CROP knowledge and innovations in terms of genetic improvement of photosynthesis and straw quality, together with straw transformation technologies could also be transferred to other major crops, first of all wheat, making the optimization of canopy photosynthesis and valorization of straw in different industrial sectors a powerful lever for the circular bioeconomy and climate change mitigation.

## CONFLICT OF INTEREST STATEMENT

The authors have not declared a conflict of interest.

## Data Availability

The data that support the findings of this study are available on request from the corresponding author. The data are not publicly available due to privacy or ethical restrictions.
